# Evaluation of ultralow‐dose computed tomography on detection of pulmonary nodules in overweight or obese adult patients

**DOI:** 10.1002/acm2.13589

**Published:** 2022-03-16

**Authors:** Xiaowan Guo, Dezhao Jia, Lei He, Xudong Jia, Danqing Zhang, Yana Dou, Shanshan Shen, Hong Ji, Shuqian Zhang, Yingmin Chen

**Affiliations:** ^1^ Department of Radiology Hebei General Hospital Xinhua District Shijiazhuang Hebei Province China; ^2^ Department of Urology The Second Hospital of Hebei Medical University Xinhua District Shijiazhuang Hebei Province China; ^3^ Siemens Healthcare Ltd. Chaoyang District Beijing China

**Keywords:** computed tomography, obesity, overweight, pulmonary nodule, radiation dosage

## Abstract

**Purpose:**

To evaluate the accuracy of pulmonary nodule (PN) detection in overweight or obese adult patients using ultralow‐dose computed tomography (ULDCT) with tin filtration at 100 kV and advanced model‐based iterative reconstruction (ADMIRE).

**Methods:**

Eighty‐one patients with body mass indices of ≥25 kg/m^2^ were enrolled. All patients underwent low‐dose chest CT (LDCT), followed by ULDCT. Two radiologists experienced in LDCT established the standard of reference (SOR) for PNs. The number, type, size, and location of PNs were identified in the SOR. Effective dose, objective image quality (IQ), and subjective IQ based on two radiologists’ scores were compared between ULDCT and LDCT. The detection performances of radiologists based on ULDCT were calculated according to the nodule analyses. Logistic regression was used to test for independent predictors of PN detection sensitivity.

**Results:**

Both the effective dose and objective IQ were lower for ULDCT than for LDCT (both *p *< 0.001). Both radiologists rated the subjective IQ of the overall IQ on ULDCT to be diagnostically sufficient. In total, 234 nodules (mean diameter, 3.4 ± 1.9 mm) were classified into 32 subsolid, 149 solid, and 53 calcified nodules according to the SOR. The overall sensitivity of ULDCT for nodule detection was 93.6%. Based on multivariate analyses, the nodule types (*p* = 0.015) and sizes (*p* = 0.013) were independent predictors of nodule detection.

**Conclusions:**

Compared with LDCT, ULDCT with tin filtration at 100 kV and ADMIRE could significantly reduce the radiation dose in overweight or obese patients while maintaining good sensitivity for nodule detection.

## 1 INTRODUCTION

Lung cancer is the most common cancer worldwide and has the highest mortality rate. Hence, early identification in small pulmonary nodules (PNs) is vital. Recently, the value of chest computed tomography (CT) as a lung cancer screening tool has been confirmed in reducing mortality in various large clinical trials.^1^, ^2^, ^3^ Therefore, chest CT is recommended in China as an optimal method for physical examinations of older patients and patients with risk factors.^4^

Radiation exposure is one limitation of chest CT. During CT, the patient is exposed to ionizing radiation.^5^ As the National Lung Screening Trial, the average estimated effective dose (ED) of one low‐dose CT (LDCT) scan is 1.5 mSv.^6^ In American College of Radiology‐Society of Thoracic Radiology (ACR‐STR) practice parameters for lung cancer screening, it is suggested that images should be optimized to avoid artifacts and provide high spatial resolution while maintaining a CT dose volume index of ≤3.0 mGy for average‐size patients, with appropriate adjustments for larger or smaller patients.^7^ Recently, one ultralow‐dose CT (ULDCT) screening protocol, which requires only 1/10 radiation dose of LDCT, can be performed using some current third‐generation, dual‐source CT scanners, and this has attracted extensive attention among radiologists.^8^, ^9^, ^10^, ^11^

However, the image quality (IQ) of ULDCT is compromised when compared with LDCT. Various advanced methods of reconstruction and postprocessing have been developed, aiming to improve the spatial resolution and IQ. Among these, the spectral shaping technique combined with advanced model‐based iterative reconstruction (ADMIRE)—which is the third‐generation of dual‐source CT—significantly reduces the radiation dose that patients are subjected to without noticeable compromises in IQ.^12^, ^13^, ^14^ For instance, Messerli et al.’s^10^ study has shown overall 91% sensitivity in detecting PN in individuals with all levels of weight. In their study, the sensitivity of ULDCT decreased with increasing body mass index (BMI). However, in their study,^10^ the tube current modulation of the scanning protocol was fixed at 70 mAs, and meanwhile the standard protocol used CARE Dose 4D which can modulate the radiation dose according to patient size and shape while producing optimal IQ. Therefore, it is still uncertain whether optimized scan protocol of ULDCT can improve the sensitivity of detecting PN in overweight and obese people.

For radiologists, keeping balance between the lower radiation dose and the good detecting of the PNs in patients classified as overweight or obese remains a challenge. A higher radiation dose is needed to lower image noise and maintain IQ in overweight or obese patients.^13^, ^15^ To minimize the radiation dose without reducing the nodule detection rate, the present study aimed to evaluate the accuracy of a ULDCT protocol with the use of the spectral shaping technique (with tin filter [TF] at 100 kV) and ADMIRE to detect PNs in patients classified as overweight or obese.

## 2 METHODS AND MATERIALS

### 2.1 Study design and subjects

**FIGURE 1:**
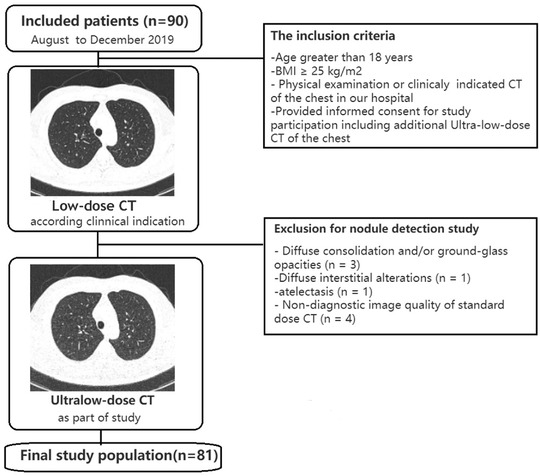
Study's flow chart

The local ethics committee approved the study, and all patients provided written informed consent to undergo LDCT and additional ULDCT. From August to December 2019, 81 patients who underwent non‐contrast‐enhanced chest CT for the initial evaluation or follow‐up of PNs were classified as overweight or obese according to the following criteria. Overweight patients were those with a BMI in the range of 25–30 kg/m^2^, whereas obese patients were those with a BMI of >30 kg/m^2^. The inclusion criteria were as follows: (1) aged >18 years, (2) BMI of ≥25 kg/m^2^, and (3) PNs <10. Patients with significant changes in the lung parenchyma on LDCT, including (1) diffuse consolidation opacities (*n* = 3) or atelectasis (*n* = 1), (2) diffuse interstitial alterations (*n* = 1), and (3) nondiagnostic IQ of LDCT dose (*n* = 2), were excluded. A flow chart of the present study is provided in Figure 1.

### 2.2 Methods

#### 2.2.1 CT examination

All examinations were performed using a third‐generation, dual‐source CT scanner (SOMATOM Force, Siemens Healthineers, Forchheim, Germany). Patients first underwent LDCT as per institutional guidelines, which was immediately followed by ULDCT. The same *z*‐axis coverage was maintained in both LDCT and ULDCT. LDCT was performed at the reference settings of 110 kV and 62 mAs with automated attenuation‐based tube current modulation (CARE Dose 4D, Siemens Healthineers) and automated attenuation‐based tube potential selection (CARE kV, setting 7, Siemens Healthineers). ULDCT was performed at a fixed tube potential of 100 kV with tin filtration and with 96 quality reference mAs using CARE Dose 4D.^16^ Other acquisition parameters were identical in the two CT scan protocols: a collimation of 96 × 0.6 mm and a slice acquisition of 192 × 0.6 mm by the means of a z‐flying focal spot were used. The gantry rotation time was 0.5 s at a pitch of 1.2, and all scans were performed during inspiratory breath hold. The ED was calculated by multiplying the dose length product (DLP) with the conversion coefficient, *k* (0.014 mSv/mGycm).^17^ The size‐specific dose estimate (SSDE) was calculated according to the American Associates of Physicists in Medicine report 204.^18^

All images were reconstructed using ADMIRE at a strength level of 3. The slice thickness was 1 mm, and a lung reconstruction kernel (BI57) and a mediastinal reconstruction kernel (BR40) were selected. The reconstructed field‐of‐view was 400 × 400 mm^2^. The image matrix was 512 × 512 pixels. All reconstructed images were transferred to the syngo.via VB10 workstation (Siemens Healthineers) for data analysis. Image analyses were performed on a high‐definition liquid crystal display monitor using the picture archiving computer‐assisted diagnosis (CAD) system (InferRead CT Lung, InferVision) and picture archiving and communication system (PACS) at our hospital. Radiologists were permitted to use all the features of the PACS at their own discretion.

#### 2.2.2 Objective and subjective evaluation of IQ

Background image noise was defined as the average of the standard deviation of the attenuation of air in the trachea above the level of the carina in three consecutive regions of interest (ROIs) at different *z*‐axis positions. One reader (5 years of experience in radiology) who was not involved in any other image evaluation work measured the image noise of LDCT and ULDCT images in a random order. The size of the ROI was 100 mm^2^ and adjacent vascular or bronchial structures were avoided. The attenuations were then measured (in Hounsfield units, HU) in the lung tissue (on the lung window image, the ROI was chosen to avoid large vessels, the trachea, and lesions), ascending aorta, and muscle (on the mediastinal window image, the ROI was chosen excluding parts of the vessel wall, calcifications, or plaques) on the same level with the same size of ROI. Signal‐to‐noise ratio (SNR) was calculated as mean CT attenuation divided by corresponding image noise.

The axial images obtained from both LDCT and ULDCT were independently presented in a random order to two readers (10 and 8 years of experience in radiology, respectively) who were blinded to all patient information and indications for CT. Images were presented with a window of −700 HU and a width of 1200 HU; readers were allowed to modify the window level and width according to their own preferences. The overall subjective IQs of LDCT and ULDCT were graded on a modified five‐point Likert scale as described previously^19^—1 point: nondiagnostic IQ, strong artifacts, score insufficient for diagnostic purposes; 2 points: severe artifacts with uncertainty about evaluation; 3 points: moderate artifacts with restricted assessment; 4 points: slight artifacts with unrestricted diagnostic image evaluation possible; and 5 points: excellent IQ with no artifacts. Scans with a quality score between 3 and 5 were considered diagnostic.

#### 2.2.3 Standard of reference

The standard of reference (SOR) was established by two radiologists (11 and 10 years of experience in radiology, respectively) who read the LDCT datasets with CAD in a random order. The radiologists were blinded to clinical information. The lesions that met the following criteria were included: (1) rounded intrapulmonary nodules according to the Fleischner Society criteria^20^; (2) nodule diameter of 1–20 mm; (3) subsolid, solid, or calcified nodules; (4) pleura‐based lesions when the center was intrapulmonary (round lesions) or the height was greater than the base (oval lesions). The following lesions were excluded: (1) perifissural nodules according to de Hoop et al.^21^ and (2) apical subpleural lesions in linear continuity with the pleura. Nodules were measured in the long‐axis and classified as either subsolid, solid, or calcified.

#### 2.2.4 Detection of PNs

Two readers evaluated ULDCT images (*n* = 81) and marked each nodule in the same way as the SOR with CAD in a second reading session. To avoid recall bias, the second reading was performed 24 weeks after the initial consensus reading. The datasets were shown in a random order to both readers, who were blinded to patient information and performed the second reading session independently; they were also unaware of the markings of the other reader. After the second reading session, every marked nodule in the ULDCT images was compared with the marked nodules in the SOR by a third radiologist (10 years of experience) side by side. This radiologist was not involved in any other image evaluation. Our ultimate goal was to find nodules accurately and comprehensively. Thus, when the results of the previous two readers differed, a final decision was reached by consensus among the three radiologists involved in this task. All correctly detected lesions, confirmed by the three readers, were classified as “true positives.” Lesions indicated on ULDCT but not on SOR were considered as “false positives.” Lesions that were not identified on ULDCT but were observed on SOR were considered as “false negatives.” For per‐patient analyses, patients with no lesions marked on CT scans (either ULDCT or SOR) were considered as “true negatives.”

### 2.3 Statistical analysis

Statistical analysis was performed with using SPSS v. 25.0 (IBM Corp.). All quantitative variables were expressed as mean ± standard deviation, and categorical variables were expressed as frequencies or percentages. The chi‐squared test was used to analyze nonparametric data, and paired sample or two‐sample *t*‐tests were used to analyze continuous variables. A two‐sided *p*‐value of <0.05 was considered statistically significant. Interobserver variability between the two readers regarding the subjective IQ was evaluated using the *κ* statistic. Nodule detection was analyzed in per‐patient (i.e., presence or absence of PNs per patient) and per‐nodule analysis, and the sensitivity and specificity of ULDCT to detect PNs was determined with a 95% confidence interval. The agreement between observers was assessed based on intraclass correlation coefficients (ICCs). Finally, the results of nodule detection were converted to a dichotomous variable, wherein “0” was defined as the nodule classified as a “true positive,” whereas “1” was defined as the nodule classified as a “false negative.” Regressors, including nodule type, size, localization, patient's BMI, patient's age, patient's sex, and image noise, were compared using multivariate analysis with logistic regression to test for independent predictors of PN detection. *p*‐Values were computed by likelihood‐ratio‐tests and Wald‐type confidence intervals.

## 3 RESULTS

### 3.1 Patient population

In total, 81 patients (52 men and 29 women) with BMIs of ≥25 kg/m^2^ (mean, 28.8 ± 3.5 kg/m^2^; range, 25.1–51.5 kg/m^2^) and a mean age of 50.8 ± 13.0 years (range, 25–78 years) were prospectively enrolled. With LDCT, a total of 234 nodules (32 subsolid, 149 solid, and 53 calcified) with a mean diameter of 3.4 ± 1.9 mm (range, 1–16 mm) were identified in 74 patients. Seven patients (8.64%) had no nodules and were only included in the evaluation of IQ. The characteristics of the patient population and nodule types are listed in Table 1.

**TABLE 1:** Characteristics of the patient population and nodule types

Variables	Patient or nodule, *n* (%) (except specific notes)
Patients	81
Male/female	52 (64.2)/29 (35.8)
Age (years), mean ± SD	50.8 ± 13.0
BMI (kg/m^2^), mean ± SD (range)	28.8 ± 3.5 (25.1–51.5)
Nodules as the SOR confirmed in LDCT	234
Nodule type	
Subsolid	32 (13.7)
Solid	149 (63.7)
Calcified	53 (22.6)
Nodule size (mm), mean ± SD (range)	3.4 ± 1.9 (1–16)
Diameter < 6	214 (91.5)
6 ≤ diameter < 8	11 (4.7)
Diameter ≥ 8	9 (3.8)

Abbreviations: BMI, body mass index; LDCT, low‐dose computed tomography; SD, standard deviation; SOR, standard of reference.

### 3.2 Radiation dose and objective IQ

All patients underwent LDCT and ULDCT as an add‐on protocol. This involved exposure to radiation at an average dose of 0.26 mSv. ULDCT images were obtained at a lower radiation dose than LDCT images: the CT dose index‐volume (CTDIvol), DLP, ED, and SSDE were all significantly lower in ULDCT than in LDCT (all *p* < 0.001).

Mean image noise of LDCT was significantly lower than that of ULDCT in the trachea, lung tissue, ascending aorta, and muscle (all *p* < 0.001). The mean attenuation of each structure yielded significant differences in the two scan protocol groups, namely lung tissue (*p* < 0.001) and muscle (*p *= 0.040). There was no statistical difference between the two groups in terms of the mean attenuation of the ascending aorta (*p* = 0.050). The SNRs of the trachea, lung tissue, ascending aorta, and muscle in the LDCT images were 20.03 ± 4.68, 13.04 ± 2.74, 0.51 ± 0.23, and 0.550 ± 0.23, respectively, which were significantly higher than those in the ULDCT images (14.07 ± 3.52, 10.18 ± 1.91, 0.37 ± 0.08, and 0.39 ± 0.10, respectively). The details of the radiation dose and assessment of objective IQ are shown in Table 2.

**TABLE 2:** Assessment of radiation dose and objective image quality in different scan protocol groups

	Low‐dose protocol	Ultralow‐dose protocol	*p‐*Value*
Radiation dose			
CTDIvol (mGy)	3.32 ± 1.10	0.52 ± 0.19	<0.001
DLP (mGy cm)	120.22 ± 44.89	18.81 ± 7.51	<0.001
ED (mSv)	1.68 ± 0.63	0.26 ± 0.11	<0.001
SSDE (mGy)	4.12 ± 1.25	0.64 ± 0.21	<0.001
Objective image quality			
Image noise			
Trachea	52.16 ± 7.34	66.70 ± 8.60	<0.001
Lung tissue	70.39 ± 12.94	88.03 ± 14.45	<0.001
Ascending aorta	93.86 ± 15.37	119.83 ± 15.20	<0.001
Muscle	96.38 ± 19.08	124.32 ± 18.09	<0.001
Mean attenuation (HU)			
Trachea	−958 ± 24.53	−935 ± 22.59	<0.001
Lung tissue	−885.26 ± 27.49	−869.56 ± 26.39	<0.001
Ascending aorta	45.81 ± 6.57	44.07 ± 7.60	0.050
Muscle	50.00 ± 8.38	48.12 ± 9.35	0.040
Signal‐to‐noise ratio			
Trachea	20.03 ± 4.68	14.07 ± 3.52	<0.001
Lung tissue	13.04 ± 2.74	10.18 ± 1.91	<0.001
Ascending aorta	0.51 ± 0.23	0.37 ± 0.08	<0.001
Muscle	0.55 ± 0.23	0.39 ± 0.10	<0.001

Abbreviations: CTDIvol, computed tomography dose index‐volume; DLP, dose length product; ED, effective dose; HU, Hounsfield units; SSDE, size‐specific dose estimate.

* *p*‐Values were calculated from paired sample *t*‐test, and *p* < 0.05 indicated a statistically significant difference.

### 3.3 Subjective IQ

**FIGURE 2:**
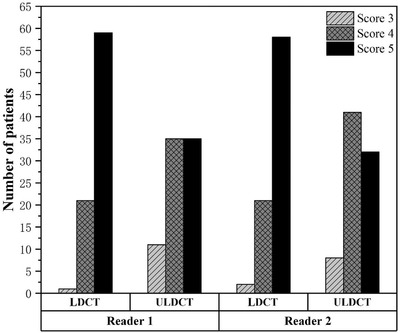
Results of subjective computed tomography (CT) image quality evaluation by the two radiologists for low‐dose and ultralow‐dose CT in 81 patients (3: moderate artifacts with restricted assessment, 4: minor artifacts, and 5: excellent image quality)

Interobserver agreement for the subjective IQ rating was better with LDCT than with ULDCT (*κ* = 0.751 vs. *κ* = 0.521, respectively). The minimum subjective IQ score for all included CT scans was 3 (moderate artifacts). This meant that the overall IQ of ULDCT was diagnostically sufficient for the 81 patients. Compared with LDCT, both radiologists rated the overall IQ with lower values for ULDCT (*p *< 0.001). Further analysis showed that radiologist 1 gave higher ratings (score 4: slight artifacts and score 5: excellent IQ) for 99% (80/81) of patients for LDCT and 86% (70/81) of patients for ULDCT (*p* < 0.001). For radiologist 2, these percentages were 98% (79/81) and 90% (73/81) (*p* > 0.001), respectively (Figure 2).

### 3.4 Detection of PNs

**FIGURE 3:**
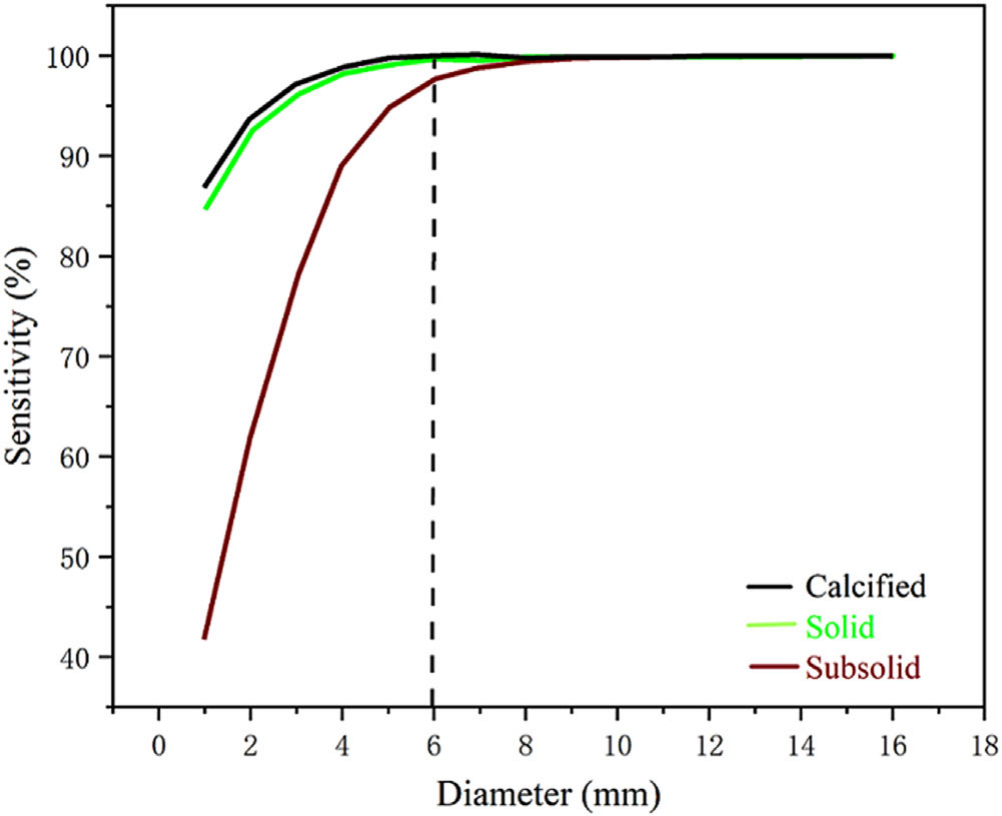
Predicted sensitivity for pulmonary nodules depending on nodule type and size in logistic regression analysis. Sensitivity increased with increasing nodule size. The predicted sensitivity for subsolid pulmonary nodules was the lowest

**FIGURE 4:**
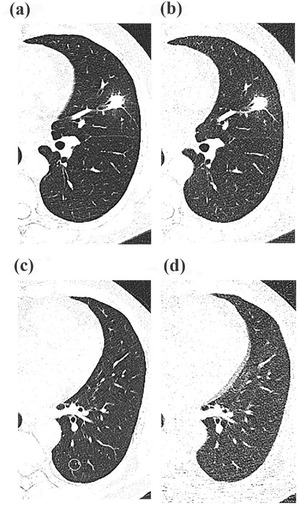
Representative transverse computed tomography (CT) sections of the lung in a 53‐year‐old man with a body mass index (BMI) of 25.7 kg/m^2^ using low‐dose CT (a) and ultralow‐dose CT (b). The solid pulmonary nodule (16 mm) in the left upper lobe was correctly detected on ultralow‐dose CT (i.e., true‐positive finding). Representative transverse CT sections of the lung in a 55‐year‐old man with a BMI of 26.1 kg/m^2^ using low‐dose CT (c) and ultralow‐dose CT (d). The solid pulmonary nodule (1 mm) in the left lower lobe was not identified on ultralow‐dose CT but was observed on the standard of reference (i.e., false‐negative finding)

**FIGURE 5:**
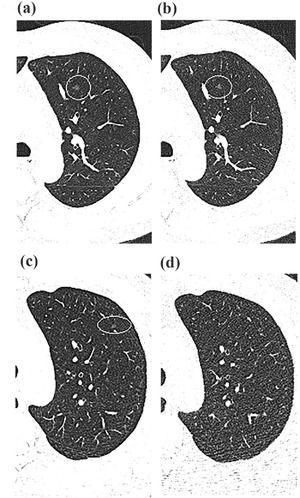
Representative transverse computed tomography (CT) sections of the lung in a 63‐year‐old man with a body mass index (BMI) of 29.7 kg/m^2^ using low‐dose CT (a) and ultralow‐dose CT (b). The subsolid pulmonary nodule (5 mm) in the left upper lobe was correctly detected on ultralow‐dose CT (i.e., true‐positive finding). Representative transverse CT sections of the lung in a 44‐year‐old man with a BMI of 26.2 kg/m^2^ using low‐dose CT (c) and ultralow‐dose CT (d). The subsolid pulmonary nodule (4 mm) in the left lower lobe was not identified on ultralow‐dose CT but was observed on the standard of reference (i.e., false‐negative finding)

There was good agreement between the two readers (ICC = 0.895) regarding PN detection. Per‐patient analysis revealed that the sensitivity, specificity, positive predictive value, and negative predictive value were 95.8%, 77.8%, 97.2%, and 70%, respectively (Table 3). On per‐nodule analysis, the overall sensitivity of PN detection was 93.6% (219/234). For solid, subsolid, and calcified nodules, the sensitivities were 95.3%, 81.3%, and 96.2%, respectively. For nodule of size <6 and ≥8 mm, the sensitivities were 93.0% and 100%, respectively (Table 4). In the present study, the univariate analysis found that variables, include age, BMI, gender, image noise, related to repeated measurement had no statistically significant impact on the correct detection of nodules. Therefore, we selected a fixed‐effects‐only model in multivariate analysis with logistic regression. The multivariate analysis revealed nodule type and nodule size to be the independent predictors of PN detection sensitivity (all *p *< 0.05) (Table 5, Figure 3). Representative cases of LDCT and ULDCT are shown in Figures 4 and 5.

**TABLE 3:** Per‐patient and per‐nodule diagnostic performance of ultralow‐dose computed tomography with low‐dose computed tomography as standard of reference

Per‐patient analysis^a^	
Number of patients	81
True positive	69 (85.2)
False negative	3 (3.7)
True negative	7 (8.6)
False positive	2 (2.5)
Sensitivity (95% CI)	95.8% (91.1%–100.6%)
Specificity (95% CI)	77.8% (43.9%–111.7%)
Per‐nodule analysis	
Number of nodules	234^b^
True positive	219 (93.6)
False negative	15 (6.4)
Sensitivity (95% CI)	93.6% (90.5%–96.7%)

*Note*: Confidence interval (CI), presented as *n* (%).

^a^ Presence or absence of pulmonary nodules per patient.

^b^ Additionally four false‐positive lesions.

**TABLE 4:** Nodule detection performance of ultralow‐dose computed tomography with low‐dose computed tomography as the standard of reference

Nodule type	Solid	Subsolid	Calcified	*p*‐Value
Number of nodules	149	32	53	
Mean diameter (mm)	3.2 ± 1.9	3.8 ± 2.0	3.4 ± 1.8	0.214^a^
True positive, *n*	142	26	51	
False negative, *n*	7	6	2	
Sensitivity (95% CI)	95.3% (91.9%–98.7%)	81.3% (67.7%–94.8%)	96.2% (91.1%–101.4%)	

Mean diameter (mm)	Diameter < 6	6 ≤ diameter < 8	Diameter ≥ 8	
Number of nodules	214	11	9	
Nodule type				0.694^b^
Solid, *n*	140 (65.4)	5 (45.5)	4 (44.4)	
Calcified, *n*	46 (21.5)	4 (18.2)	3 (33.3)	
Subsolid, *n*	28 (13.1)	2 (18.2)	2 (22.2)	
True positive, *n*	199	11	9	
False negative, *n*	15	0	0	
Sensitivity (95% CI)	93.0% (89.6%–96.4%)	100%	100%	

*Note*: Confidence interval (CI), presented as *n* (%) and mean ± standard deviation (range).

^a^ Wilcoxon Mann–Whitney test.

^b^ Chi‐squared test.

**TABLE 5:** Univariate and multivariate analyses of nodule diagnostic performances of ultralow‐dose computed tomography with low‐dose computed tomography as standard of reference

	Univariate analysis				Multivariate analysis with logistic regression	
Variables	Total (%) (*n* = 234)	Detected (%) (*n* = 219)	Not detect (%) (*n* = 15)	*p*‐Value	Odds ratio (95% CI)	*p*‐Value^e^
Nodule characteristics						
Nodule type				0.019^a^		0.015
Solid	149 (63.7)	142 (64.8)	7 (46.7)		Reference	
Subsolid	32 (13.7)	26 (11.9)	6 (40.0)		7.17 (1.80, 28.59)	
Calcified	53 (22.6)	51 (23.3)	2 (13.3)		0.86 (0.17, 4.18)	
Nodule size (mm)	3.4 ± 1.9	3.4 ± 1.9	2.4 ± 1.5	0.018^b^	0.46 (0.25, 0.85)	0.013
Nodule localization				1.000^a^		0.954
Upper lobe	99 (42.3)	92 (92.9)	7 (7.1)		Reference	
Middle lobe	37 (15.8)	35 (94.6)	2 (5.4)		1.25 (0.22, 7.08)	
Lower lobe	98 (41.9)	92 (93.9)	6 (6.1)		1.77 (0.33, 4.18)	
Clinical characteristics						
BMI (kg/m^2^)	28.8 ± 3.5	28.8 ± 3.5	29.7 ± 3.7	0.431^b^	1(0.86, 1.17)	1.000
Age (years)	52.6 ± 11.9	53.7 ± 11.1	52.6 ± 11.3	0.725^c^	1.01 (0.86,1.17)	0.772
Gender				0.106^d^		0.415
Male	154 (65.8)	147 (67.1)	7 (46.7)		Reference	
Female	80 (34.2)	72 (32.9)	8 (53.3)		1.65 (0.51, 5.39)	
Image noise (trachea) (HU)	66.6 ± 9.0	66.4 ± 8.0	69.5 ± 8.2	0.194^c^	1.04 (0.96, 1.14)	0.335

*Note*: Data are presented as odds ratio (95% confidence interval, CI), *n* (%), or mean ± standard deviation (range).

Abbreviations: BMI, body mass index; HU, Hounsfield units.

^a^ Likelihood‐ratio‐tests.

^b^ Fisher chi‐squared test.

^c^ Wilcoxon Mann–Whitney test.

^d^ Independent‐sample *t*‐test.

^e^ Chi‐squared test.

## 4 DISCUSSION

It is generally believed that IQ decreases with the decrease in radiation dose. Therefore, the present study compared the radiation dose and IQ between ULDCT and LDCT among patients who were overweight or obese. The mean ED dose of ULDCT was significantly lower than that of LDCT in this study. However, all the ULDCT images had diagnostically acceptable IQ, consistent with the findings of some previous studies.^10^, ^12^, ^22^ We thought that this was attributed to the advance CT scan and reconstruction technology used in the present study. First, the optimization in the tube voltage in combination with TF was adopted to reduce patient dose in this study. This technique has been established particularly for contrast‐enhanced CT and is considered to be of no benefit for non‐contrast CT. However, there is an optimal energy level for obtaining data, which leads to a reduction in the radiation dose to the patient.^23^, ^24^ Dual‐energy imaging, which is achieved by an improved separation of the two energy spectra and dose‐neutral scanning, also applies (and derives) the same principle. By adding TF, low‐energy photons that contributed little to IQ but increased the radiation dose were primarily absorbed. This reduces the radiation dose for low‐tube current protocols while maintaining a good IQ as fewer photons traverse the patient.^16^ Second, our study was performed with ADMIRE at a strength level of 3. This advanced algorithm technique has been proved to further improve the spatial resolution and IQ in chest imaging.^25^, ^26^, ^27^, ^28^ Therefore, our study demonstrated that ULDCT with a TF of 100 kV and ADMIRE used in overweight or obese patients can maintain a diagnostically acceptable IQ.

Furthermore, with diagnostically acceptable IQ, the present study evaluated the accuracy of ULDCT for PN detection in overweight or obese patients. The sensitivity of ULDCT for PN detection was good in the present study. However, both univariate and multivariate analyses showed that the patients’ BMIs had no significant effects on the detection rates of solids and subsolid PNs, which was inconsistent with the findings of a previous study.^10^ We attribute this to the differences in the BMIs of the cohorts and the differences in the scanning protocols. In the previous study,^10^ the range of the patients’ BMIs was larger (mean ± standard deviation [range], 26.2 ± 5.3 kg/m^2^ [15.9–49 kg/m^2^]), and almost all levels of the BMI subgroups were covered; hence, this study concluded that the patients’ BMIs had significant effects on the PN detection rate. However, the present study aimed to investigate the PN detection rate in overweight or obese patients, and only patients classified as overweight or obese were included. Therefore, the BMI range in this study was relatively limited. In addition, most enrolled patients (nearly 80%) had BMIs of 27–30 kg/m^2^, and only few had BMIs >30 or <27 kg/m^2^. Hence, it is possible that there was not enough data to detect the effect of the patients’ BMIs on the PN detection rate. Moreover, we used the adaptive tube current modulation technology of third‐generation dual‐source CT, which was not used in the previous study.^10^ We hope that future studies confirm this conclusion by including more patients with a BMI of >30 kg/m^2^.

Our study demonstrated that nodule types and nodule sizes were independent predictors of PN detection sensitivity in patients classified as overweight or obese. In the present study, the sensitivity of PN detection increased markedly with the increase in nodule diameter, and the effect differed across the three nodule types (Figure 3). The overall sensitivity for subsolid nodules was lower than that for the other two nodule types, which can be explained by lower attenuation, consistent with prior studies.^9^, ^10^, ^29^, ^30^ When evaluating subsolid nodules with diameters of ≥6 mm, which need a follow‐up according to the recommendations of the Fleischner Society,^31^ the sensitivity was 100% in the present study. However, the data for subsolid nodules were limited. Therefore, we believe that ULDCT can meet the sensitivity requirement for PN detection in clinical practice, and future studies with a larger number of subsolid nodules are warranted to evaluate the value of this CT protocol for this type of nodule.

There were four false‐positive nodules in two patients in the present study. We assumed that it may result from volume artifacts and higher noise from ULDCT. First, on ULDCT, three of them appeared too close to the bronchovascular bundle and one appeared close to the pleura; their location distributions made these areas prone to the volume effect of adjacent structures. In addition, the increased noise in ULDCT, combined with the volume effect, further increases the likelihood of false positives. However, the false‐positive rate was as low as 1.7%, and the maximum diameter of these four nodules was approximately 3 mm in the present study. According to the Fleischner Society^31^ and the National Comprehensive Cancer Network (NCCN) guidelines,^32^ the risk for malignancy of nodules smaller than 3 mm is very low. Hence, no routine follow‐up was recommended for these nodules, and it will not affect the follow‐up strategy of PN screeners.

There were several limitations in the present study. First, the sample size was relatively small, and nodules with diameters of <3 mm were included in the present study. However, the purpose of the present study was to evaluate the sensitivity and independent predictors of PN detection of the ULDCT protocol. Second, we did not reconstruct images at varying strength levels of ADMIRE, because of which the potential effects of reconstruction parameters on nodule detectability could not be evaluated in the present study. Third, we did not test the intraobserver variability between the readers in detecting PNs for the ULDCT protocol. Fourth, logistic regression with only fixed effects was used in the present study.

## 5 CONCLUSION

Compared with LDCT, ULDCT with TF at 100 kV and ADMIRE could significantly reduce the radiation dose in overweight or obese patients. On per‐nodule analysis, ULDCT could maintain a good sensitivity for PN detection.

## CONFLICTS OF INTEREST

This research did not receive any specific grant from funding agencies in the public, commercial, or not‐for‐profit sectors.

## AUTHOR CONTRIBUTIONS

Xiaowan Guo assisted in conceptualization, methodology, data curation, writing—original draft preparation, and software. Dezhao Jia, Lei He, Xudong Jia, and Danqing Zhang assisted in data curation and software. Yana Dou NY, Shanshan Shen, and Hong Ji assisted in software. Yingmin Chen and Shuqian Zhang assisted in supervision, writing—review and editing. All authors read and approved the final manuscript.
